# Structural and functional insights into the mechanism of action of plant borate transporters

**DOI:** 10.1038/s41598-021-91763-6

**Published:** 2021-06-10

**Authors:** Savvas Saouros, Thotegowdanapalya C. Mohan, Cristina Cecchetti, Silke Lehmann, Joseph D. Barrit, Nicola J. Scull, Paul Simpson, Yilmaz Alguel, Alexander D. Cameron, Alexandra M. E. Jones, Bernadette Byrne

**Affiliations:** 1 Department of Life Sciences, Imperial College London, Exhibition Road, London, SW7 2AZ UK; 2 School of Life Sciences, University of Warwick, Gibbet Hill Road, Coventry, CV4 7AL UK; 3 Present Address: Department of Biotechnology and Bioinformatics, Faculty of Life Sciences, JSS Academy of Higher Education and Research, Mysore, Karnataka 570015 India

**Keywords:** Membrane proteins, Cryoelectron microscopy, Plant physiology

## Abstract

Boron has essential roles in plant growth and development. BOR proteins are key in the active uptake and distribution of boron, and regulation of intracellular boron concentrations. However, their mechanism of action remains poorly studied. BOR proteins are homologues of the human SLC4 family of transporters, which includes well studied mammalian transporters such as the human Anion Exchanger 1 (hAE1). Here we generated *Arabidopsis thaliana* BOR1 (AtBOR1) variants based (i) on known disease causing mutations of hAE1 (S466R, A500R) and (ii) a loss of function mutation (D311A) identified in the yeast BOR protein, ScBOR1p. The AtBOR1 variants express in yeast and localise to the plasma membrane, although both S466R and A500R exhibit lower expression than the WT AtBOR1 and D311A. The D311A, S466R and A500R mutations result in a loss of borate efflux activity in a yeast *bor1p* knockout strain. *A. thaliana* plants containing these three individual mutations exhibit substantially decreased growth phenotypes in soil under conditions of low boron. These data confirm an important role for D311 in the function of the protein and show that mutations equivalent to disease-causing mutations in hAE1 have major effects in AtBOR1. We also obtained a low resolution cryo-EM structure of a BOR protein from *Oryza sativa*, OsBOR3, lacking the 30 C-terminal amino acid residues. This structure confirms the gate and core domain organisation previously observed for related proteins, and is strongly suggestive of an inward facing conformation.

## Introduction

Boron (B) is a chemical element key for plant growth and development, playing essential roles in formation and stability of the plant cell wall and is crucial for normal leaf expansion, through the cross-linking of the polysaccharide rhamnogalacturonan II (RG-II)^1^. Borate-diol diester bridges form between the apiosyl residues of two monomers of RG-II^2^. Boron deficiency or excess causes a range of plant growth defects^3–5^ and is a major issue for crop growth^6^. The optimal B concentration is narrow in many plants; in wheat it is between 10 and 100 µg/g tissue concentration^7^. B is highly soluble and thus is readily leached from the soil in areas with high rainfall including parts of the USA and China^8^. The resulting B deficiency limits plant growth, root elongation, fruit ripening and seed production^9^. Conversely excess B is often the result of low rainfall in areas of, for example, Australia, Turkey and South America and the Middle East and reduces root cell division and chlorophyll content, which in turn limits growth of shoots and roots^10^.

Plants have a complex system of channels and transporters involved in the uptake and distribution of boron^11^. Boron is taken up from the soil in the form of boric acid, B(OH_3_), via a passive process, through a combination of direct diffusion across the membrane and facilitated diffusion through the nodulin 26-like intrinsic proteins (NIPs)^12^. Borate transporters (BORs) are responsible for active efflux of boron from cells, most likely in the charged borate [B(OH_4_)]^−^ form^8^. The BORs are thus suggested to be borate antiporters coupling efflux of the substrate to the co-transport of a H^+^^13^. The first identified and best characterised BOR protein, BOR1 from *Arabidopsis thaliana* (AtBOR1), is responsible for active export of B from the root cells to the xylem for further distribution around the plant^14^. Overexpression of AtBOR1 leads to more efficient uptake of B and resistance to B deficiency^15^. BOR proteins are also key in preventing toxic accumulation of B in cells. Increased levels of AtBOR4 from *A. thaliana*^16^ and the homologue Bot1 from barley^17^ confer increased tolerance of high B concentrations by exporting the excess B back into the soil. AtBOR1 is regulated at least in part by cellular B concentration, with low B concentration resulting in increased levels of the transporter at the membrane and high B concentrations resulting in the transporter being targeted for degradation^18^.

Plants have been shown to exhibit differences in their sensitivities to B deficiency and in the patterns of expression of BOR proteins. *A. thaliana* has previously been reported to succumb to B deficiency at levels less than 0.5 μM B, whereas rice (*Oryza sativa*) is only sensitive at levels lower than 0.2 μM B indicating that *O. sativa* is more tolerant to B deficiency^19^. In *A. thaliana,* BOR1 is expressed in the root endodermis. Whereas BOR1 from *Oryza sativa* (rice), OsBOR1, is expressed in both the endodermis/stele (for xylem loading) and in the exodermis under conditions of B deficiency. However, under conditions of B sufficiency OsBOR1 expression is only detectable in the stele^20^. In both species BOR1 allows passage of B though the Casparian strips thus enabling active efflux of B from the root cells to the xylem^20^.

BOR proteins share homology with the Solute Carrier (SLC) 4 family of secondary active transporters, that includes proteins which function as either symporters or exchangers^21^. Four structures of proteins from the SLC4 family, and homologues, are available: the human Anion Exchanger 1 (hAE1)^22^, the acid–base transporter NBCe1^23^, *Saccharomyces mikatae* Bor1p^24^ and *Arabidopsis thaliana* BOR1 (AtBOR1)^25^. The structures revealed a protomer arrangement of 7 + 7 transmembrane (TM) segments in an inverted repeat architecture. Each protomer is comprised of two different domains, the core and the gate, responsible for substrate transport and dimerization respectively. The SLC4 proteins are structurally related to the SLC26 family including the structurally characterised SLC26Dg^26,27^ and BicA^28^, as well as the SLC23 family including structurally characterised UapA^29^ and UraA^30^. All of these proteins are dimeric and suggested to function via an elevator mechanism where substrate binds into a site formed by two half helices, TMs3 and 10, within the core domain and this domain then moves relative to the gate domain pulling the substrate across the membrane^31,32^. In the case of UapA and UraA it is clear that the dimer is critical for function whereas the role of the dimer in the other proteins is less defined. Research on the *S. cerevisiae* homologue, ScBOR1p, supported the dimer as the oligomeric form of the protein and found that dimer formation was dependent upon the presence of membrane lipids, but also showed that the protein could function as a monomer^33^.

Despite progress in understanding of the structure and function of the BOR proteins, many questions remain. Here we used a combined structural and functional approach including plant phenotypes to explore the AtBOR1 and OsBOR3 proteins. Our research shows that the equivalent of disease-causing mutations from hAE1 also have highly detrimental effects on BOR protein function and plant growth. Specifically mutations G796R^34^) and S762R^35^ in hAE1 abolish anion exchange and cause stomatocytosis. The equivalent mutations in AtBOR1, A500R and S466R respectively, cause reduced borate efflux leading to boron deficiency symptoms in plants including reduced plant weight. The same results are seen for a mutant of D311, a possible proton binding residue. A low resolution cryo-EM structure of OsBOR3 provides the first glimpse of the molecular details of a rice borate transporter.

## Results

### Mutations on AtBOR1 were selected based on homology with hAE1

**Figure 1:**
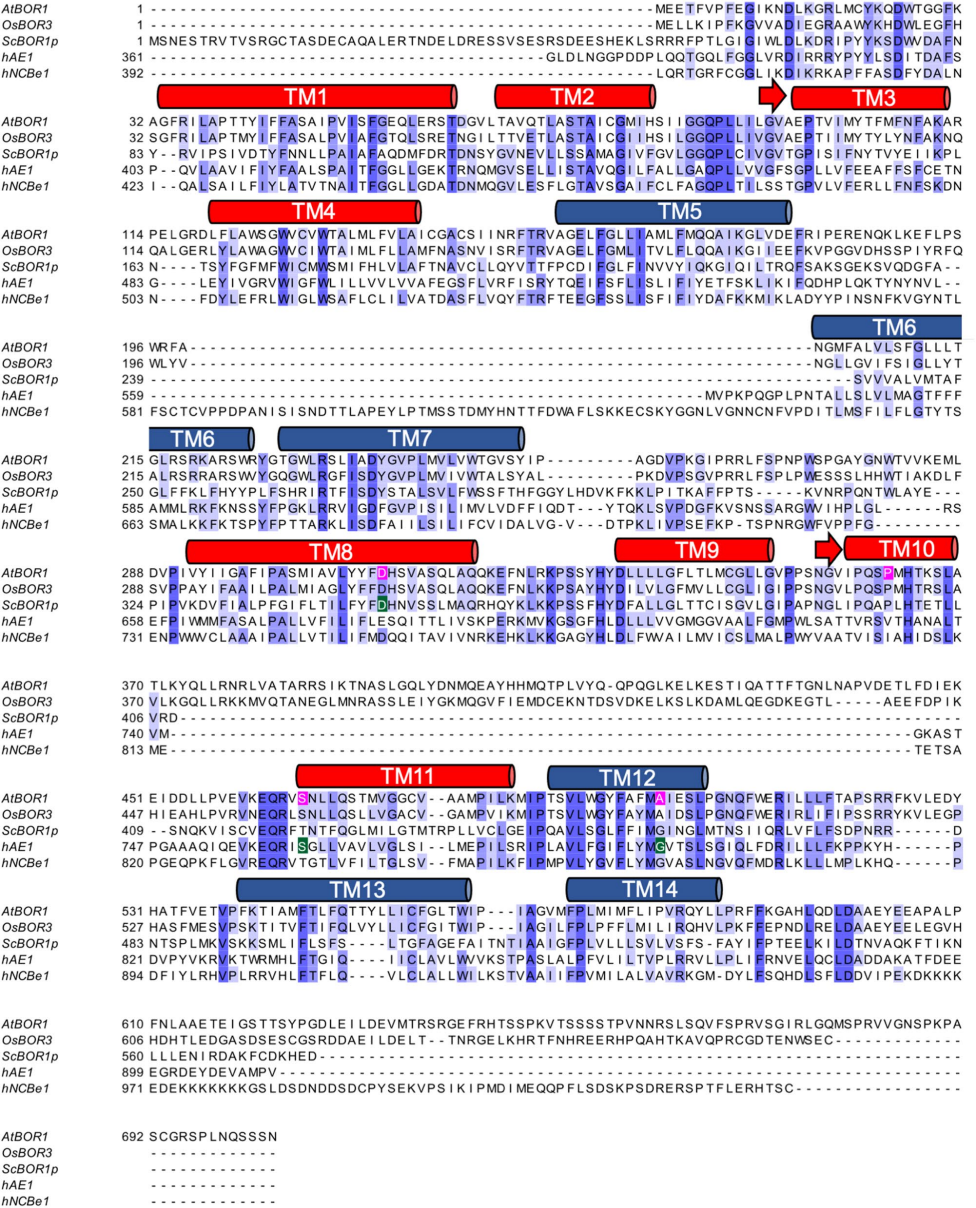
Protein sequence alignment of SLC4 family members. AtBOR1 (Acc. Code: OAP07993.1), OsBOR3 (Acc. Code: AK072421), hAE1 (Acc. Code: NP_000333.1), hNBCe1 (Acc Code: NP_003750.1). Only the TM domains of hAE1 and hNBCe1 are aligned with the full length plant BOR proteins. The approximate positions of the TM domains based on the X-ray crystal structure of AtBOR1 (PDB 5L25) are indicated in the cylinders above the sequence. The TM domains are coloured with the helices comprising the core domain in red and the helices comprising the gate domain in blue. The positions of the single point mutations introduced in AtBOR1 are indicated in magenta.

D311 (Fig. 1) is a possible candidate H^+^ binding residue and mutation of the equivalent residue (D347) in *S. cerevisiae* has previously been shown to inhibit borate efflux function in a yeast based functional complementation assay^25^. As described above, S466R (equivalent to S762R^35^) and A500R (equivalent to G796R^34^) are equivalent to disease causing mutations in hAE1. In the context of the hAE1 structure, these mutations cannot be accommodated without modification to the overall fold^22^. The mutant P362G, which was generated as part of another study, had no effect on AtBOR1 transporter function (see “Results” section) and is included here as a control for the generation of the plant variants.

### The AtBOR1 variants localise to the plasma membrane in yeast but exhibit varying stability in detergent based solution

**Figure 2:**
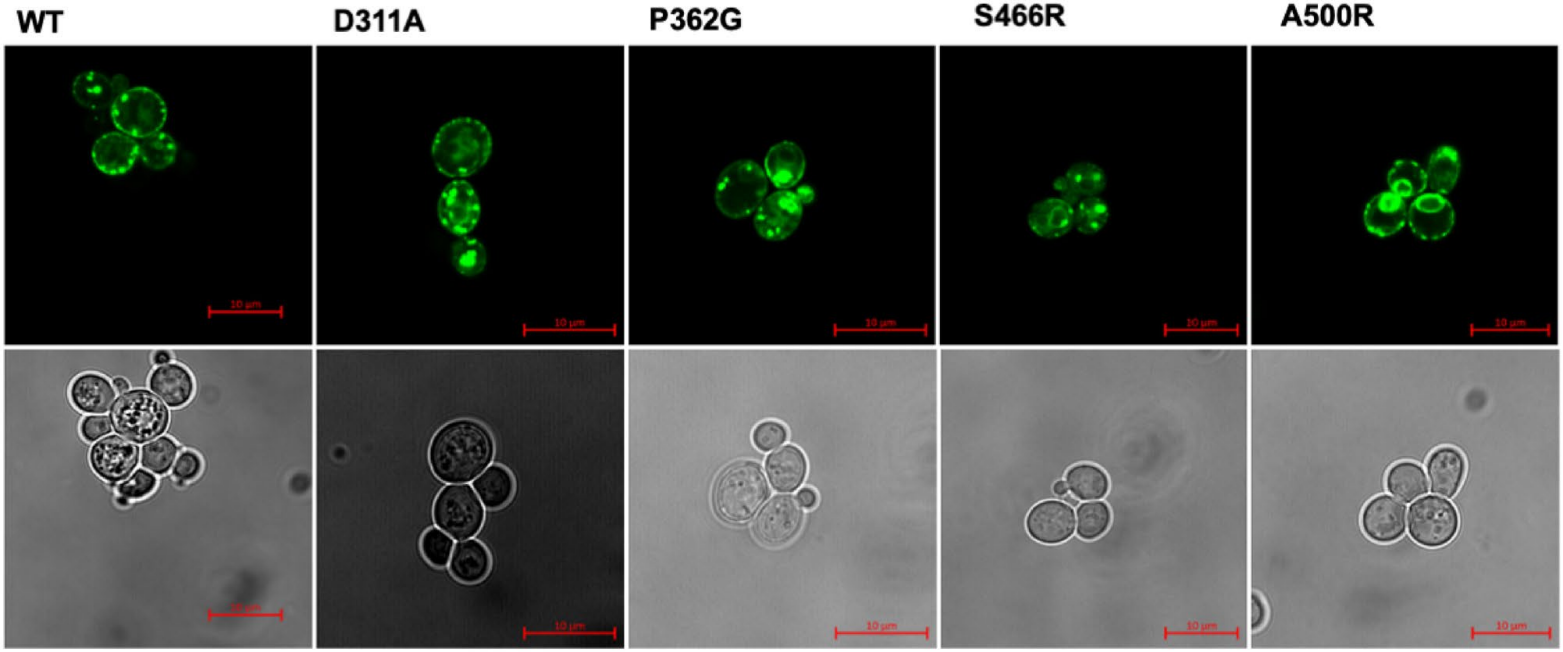
Localisation of AtBOR1-GFP variants in yeast strain FGY217. The proteins were visualised using both confocal fluorescence microscopy for detection of GFP (upper panel) and corresponding bright field images (lower panel). Data shown is representative of at least n = 2 independent experiments. Scale bar = 10 μm.

**Figure 3:**
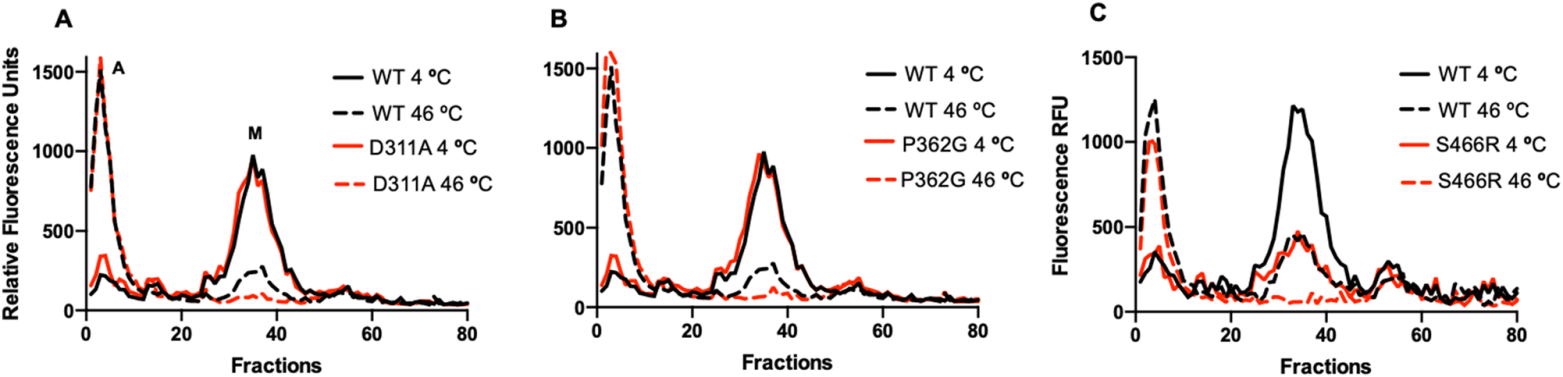
FSEC and hFSEC analysis of AtBOR1 proteins. FSEC analysis is carried out on protein solubilised at 4 °C prior to separation on the SEC column and hFSEC analysis is carried out on protein initially prepared in the same way then heated at 46 °C for 10 min, prior to separation on the SEC column. In each case, aggregated material elutes at roughly fraction 8, with monodispersed protein eluting at roughly fraction 35, labelled A and M respectively on (**A**). (**A**–**C**) Show the results obtained for D311A, P362G and S466R variants compared to WT AtBOR1 analysed at the same time, respectively. In each case the elution trace obtained for unheated sample is shown in the solid line while the elution trace obtained for heated sample is shown in the same coloured dashed line. Data is representative of at least n = 2 independent experiments.

All variants had similar expression levels in yeast to WT AtBOR1 (range 1.7–3 mg/L) with the exception of the A500R mutant protein which exhibited markedly lower expression (0.7 mg/L, Supplementary Table S2). All proteins traffic to the plasma membrane in yeast (Fig. 2), however in all cases there is a high level of fluorescent material clearly visible within the cells. It is likely that this is incorrectly folded material that is retained within the ER and ultimately targeted for degradation^36^. In order to determine the stability of the AtBOR1 variants we used fluorescent size exclusion chromatography (FSEC) and heated FSEC (hFSEC). All proteins solubilised into detergent (DDM)-based solution with similar solubilisation efficiencies (50–80%, Supplementary Table S2), although the amount of monodispersed protein differs as a result of variations in the protein expression level, particularly with respect to S466R (Fig. 3; Supplementary Table S2). The exception was A500R where the amounts of protein extracted into detergent (29%, Supplementary Table S2) were too low to be detectable following separation on the SEC column. In order to further characterise the individual proteins we also submitted them to hFSEC, heating the solubilised proteins to 46 °C (higher than the apparent T_m_ for WT AtBOR1 fused with GFP^37^) for 10 min prior to separation on the SEC column. Following heating the WT monodispersed protein peak height is substantially reduced with a concomitant increase in the size of the aggregation peak. There was no detectable monodisperse S466R protein after heating, indicating that this protein is much less stable than the WT AtBOR1. In contrast, the D311A and P362G proteins gave very similar results to the WT protein following heating (Fig. 3).

### Borate efflux was disrupted by mutations D311A, S466R and A500R

**Figure 4:**
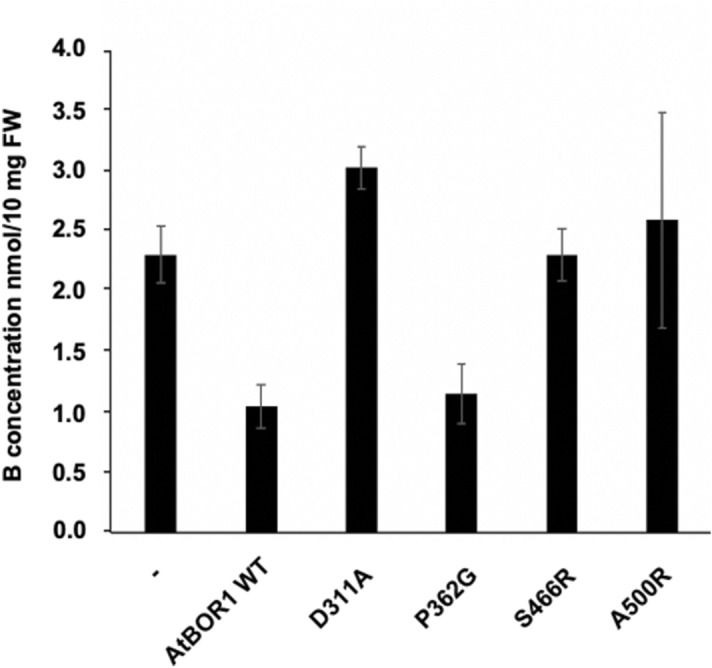
Borate efflux activity of the AtBOR1 variants in yeast. FGY217 Δ*bor1p* cells expressing each of the AtBOR1 proteins were assessed for their ability to export boron relative to control cells transformed with vector only. −  = vector only control. Intracellular B content (nmol/10 mg FW) after 60 min incubation of cells in 1 mM boric acid. Values have been normalised to GFP concentration. Data shown is mean ± S.D. of n = 3 independent experiments.

The borate efflux activity of all the proteins was assessed by expression in a ∆*bor*1p strain. Yeast cells expressing both WT AtBOR1 and P362G exhibit reduced levels of intracellular boron compared to cells containing a vector only control (Fig. 4). D311A in contrast exhibits a similar level of intracellular boron to the vector only control indicating no discernable borate efflux activity and in agreement with the results obtained for the ScBOR1p equivalent mutant (D347^25^). Yeast cells expressing the S466R and A500R are also unable to export excess boron with similar levels of intracellular boron to the vector only control.

### AtBOR1 variants affect plant growth

**Figure 5:**
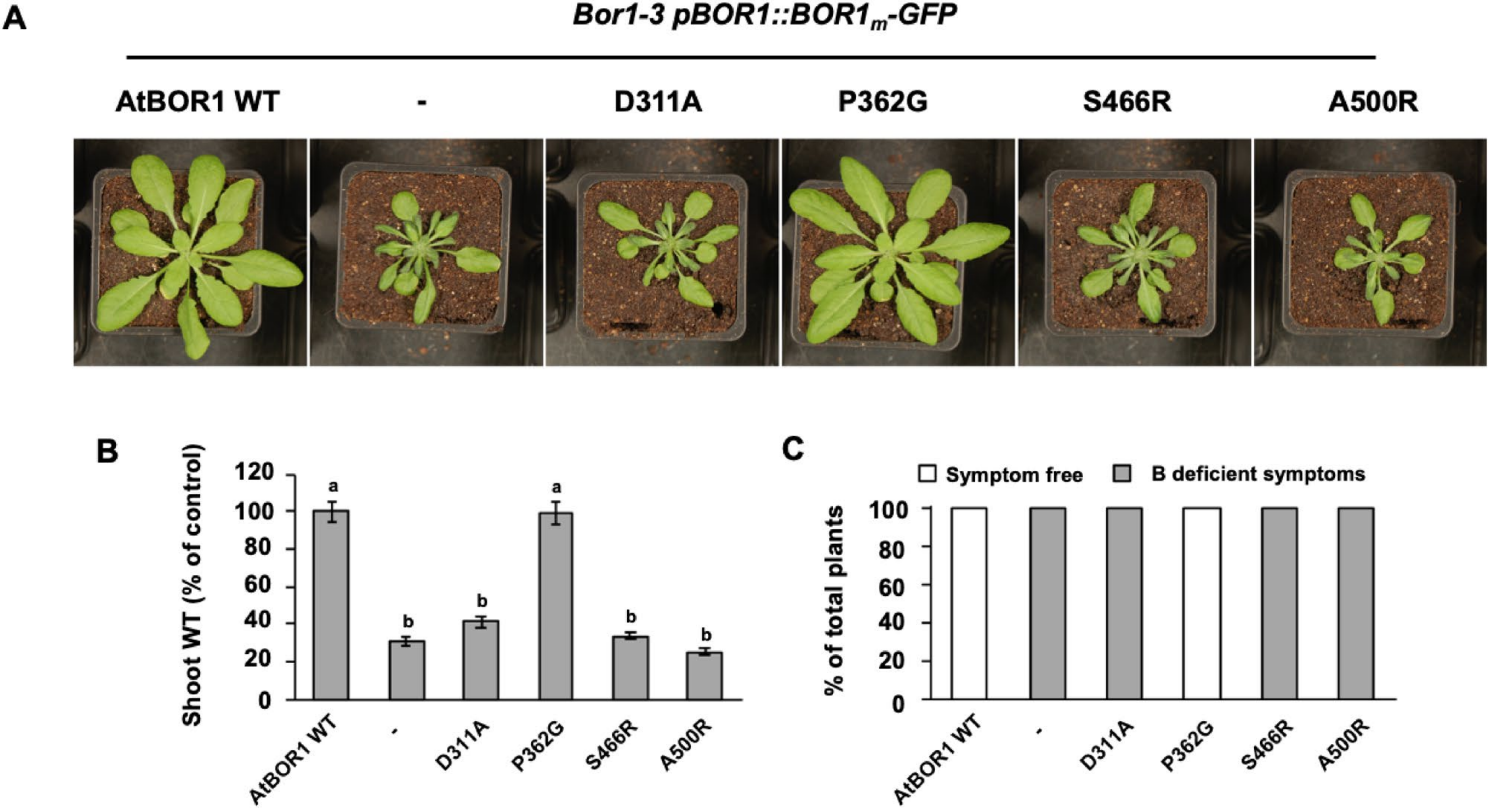
Phenotypes of different AtBOR1 variant plants grown in soil. The negative (−) control is the *bor1-3* KO plant. (**A**) Images of the different plants grown in soil without addition of boron. (**B**) Comparison of the fresh weight of the shoots of the different AtBOR1 variant plants, a, p < 0.01 compared to the negative control of the *bor1-3* KO plants, b, p < 0.01 compared to WT AtBOR1 (ANOVA followed by Tukey’s test). Data is expressed relative to WT AtBOR1 and is mean ± S.E. of n ≥ 9 independent plants. (**C**) Overall % of plants exhibiting either no B deficiency (white) or B deficiency (grey) for each of the tested variants. Data is representative of 3 independent experiments.

When expressed as C-terminal GFP fusion proteins in *bor1-3*, only P362G restores shoot fresh weight and rescues B-deficiency symptoms, similar to WT BOR1 in soil-grown plants. In agreement with the results of the yeast B efflux assays D311A, S466R and A500R-expressing plants exhibit reduced shoot fresh weight and typical B-deficiency symptoms comparable to the uncomplemented *bor1-3* KO (Fig. 5).

### Variability of B deficiency phenotypes in planta

**Figure 6:**
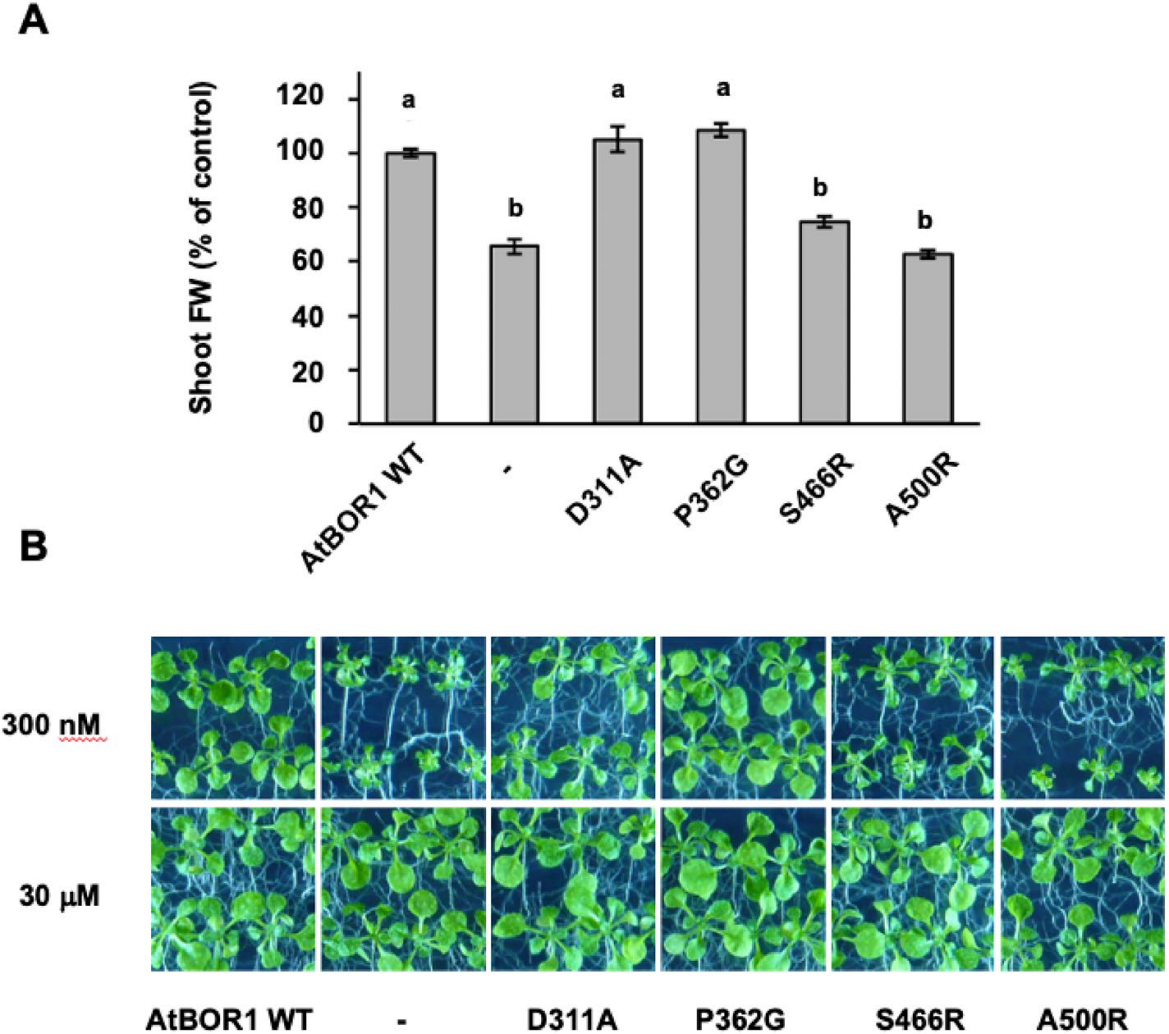
Phenotypes of AtBOR1 variant plants grown in vitro. (**A**) Comparison of the fresh weight of the different AtBOR1 variant plants grown on plates containing 300 nM boron, *a*, p < 0.01 compared to the negative control of the *bor1-3* KO plants, *b*, p < 0.01 compared to WT AtBOR1 (ANOVA followed by Tukey’s test). Data is expressed relative to WT AtBOR1 and is mean ± S.E. of n = 12 plants. (**B**) Images of AtBOR1 variant plants grown on plants on 300 nM boron (upper panels) or 30 μM boron (lower panels). Data is representative of n = 2 independent experiments.

We observed that the B deficiency phenotype of D311A in soil grown plants appeared to be variable (possibly due to watering regime, humidity or soil batches). To better control boron concentration, we grew *A. thaliana* seedlings on agar plates supplemented with boric acid. When grown axenically on medium with low boron concentration (300 nM), seedlings of *bor1-3* plants expressing S466R and A500R exhibit reduced shoot weight similar to uncomplemented *bor1-3* plants, while P362G and WT BOR1 can restore normal shoot development under B-deficiency conditions (Fig. 6). Unexpectedly, compared to the soil growth assay, shoot fresh weight of seedlings expressing D311A were not significantly different to WT AtBOR1 at 300 nM boron, whereas in the previous soil assay plants expressing D311A showed deficiency symptoms similar to *bor1-3*. These unexpected D311A phenotypes were consistent over three independent lines and several replicate experiments (of both assays). We consider that physiological parameters such as transpiration and photosynthesis rates as well as nutrient transport will differ in soil and in plate assays as a result of plant growth conditions and plant age and this might influence the development of B deficiency symptoms. The observed shoot phenotypes for the S466R and A500R variants are a result of low B supply, since all tested mutants develop similar to control plants when grown on sufficient B levels (30 µM).

### OsBOR3 is a dimer, with each protomer organised into two discrete domains

**Figure 7:**
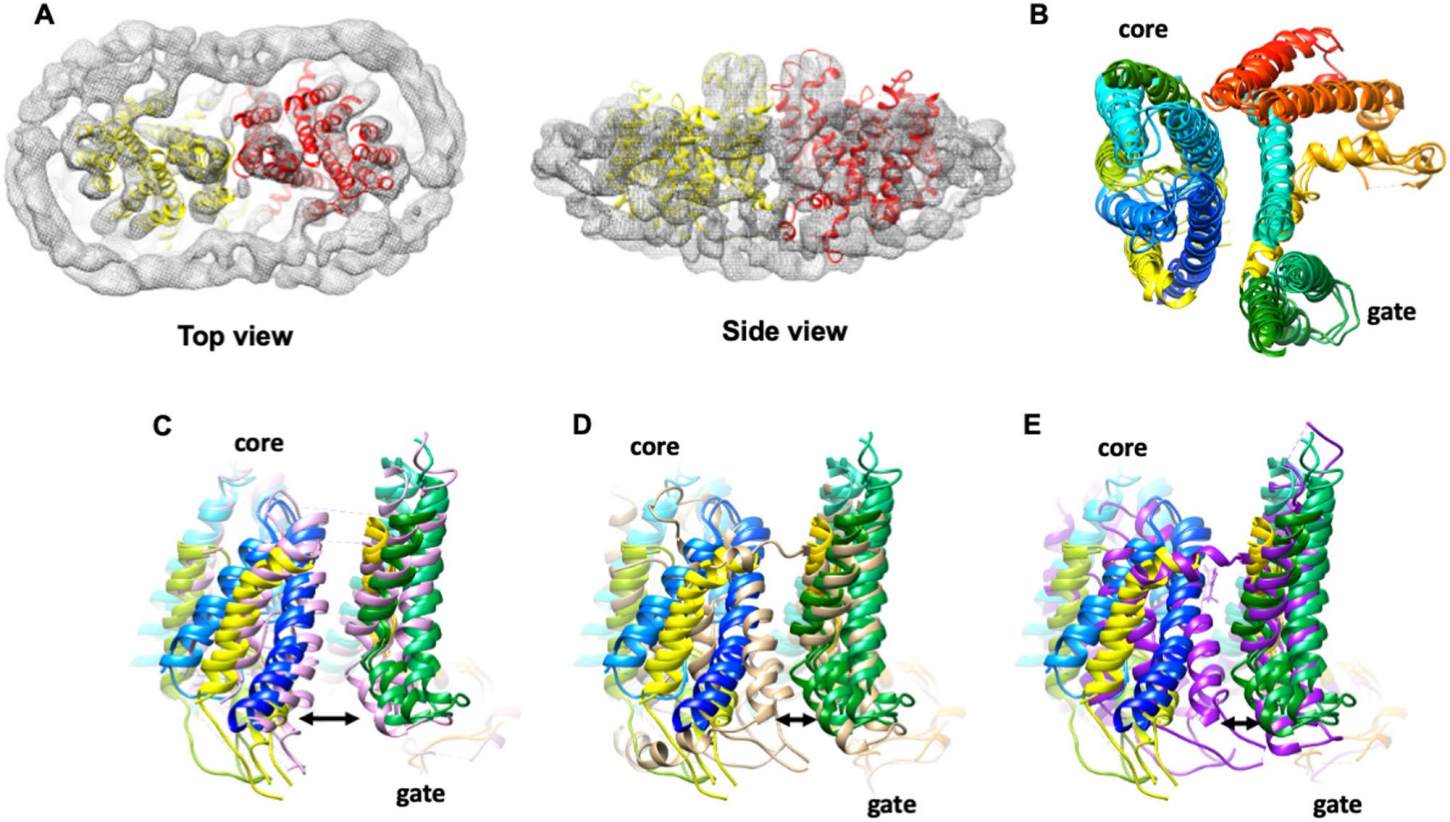
Cryo-EM structure of OsBOR3. (**A**) CryoEM density map for OsBOR3 shown from the extracellular side of the membrane and the side view looking through the membrane. The dimeric structure of AtBOR1 (individual protomers shown in yellow and red) has been fitted into the OsBOR3 EM density. The additional density around the TM domains corresponds to the LMNG detergent micelle. (**B**) The respective core and gate domains of the AtBOR1, hNBCe1 and hAE1 monomers fitted into the EM density of OsBOR3 based on the correlation coefficient in Chimera^48^. A single subunit of each protein was first fitted into the density and then the automatic fitting algorithm in Chimera was used to optimise the fit of the core and gate domains into the density separately. Each protein has been coloured in the same way with blue at the N-terminus through to red at the C-terminus. This image reveals the relative positions of the core and gate domains in the OsBOR3. Superposition of the monomeric AtBOR1 (pink, (**C**)), hNBCe1 (beige, (**D**)) and hAE1 (purple, (**E**)) onto the composite image shown in (**B**) revealing the OsBOR3 has a conformation most similar to the inward occluded AtBOR1. The images are shown from the side of the protein, looking through the membrane with the extracellular regions of the proteins at the top. The arrows highlight the more open nature of the OsBOR3 on the intracellular side of the membrane compared to hNBCe1 and hAE1.

There is a relatively low resolution (4.2 Å) X-ray crystallographic structure of a C-terminally truncated AtBOR1^25^ in the occluded conformation (closed to both sides of the membrane). In an effort to obtain additional information about the structure and mechanism of the BOR proteins we expressed and isolated a rice homologue, OsBOR3, which shares 56% sequence identity with AtBOR1 and OsBOR1 (Fig. 1). We did attempt to express and purify other rice BOR proteins, including OsBOR1, however OsBOR3 was by far the most suitable protein in terms of expression yield and stability in detergent based solution. The protein was engineered to remove the 30 C-terminal residues in order to increase stability and introduce a Q228T mutation to remove a naturally occurring TEV cleavage site to yield OsBOR3_∆1–642_. Functional complementation in yeast confirmed that this engineered version of the protein is functional (Supplementary Fig. S1A). As reported previously we were able to obtain high quality OsBOR3_∆1–642_ protein suitable for structural analysis^38^ (Supplementary Fig. S1B). The protein yielded high quality negative stain (Supplementary Fig. S1C) and cryo-EM grids (Supplementary Fig. S2A). 2D class averages were obtained of various views of the protein from a total of 116,140 particles (Supplementary Fig. S2B–D). These were used to reconstruct a 3D model with a local resolution ranging from 5 to 6 Å (Fig. 7A). As expected from our previous analysis the protein is a dimer in LMNG micelles^38^. The density map for the dimer is shown in Fig. 7A with the crystal structure of AtBOR1 fitted. We carried out separate fitting of the gate and core domain structures of outward facing hNBCe1, outward facing hAE1 and inward occluded AtBOR1 (Fig. 7B). As can be seen, despite the different conformational states, the domain structures of the different proteins converge on the OsBOR3 density confirming the organisation of the individual TMs into gate and core domains.

The core domain is less well resolved than the gate domain, probably as a result of the inherent flexibility of this region of the protein, associated with its elevator motion through the membrane. The gate domain forms the dimerization interface and as a result is much more static (Supplementary Fig. S3).

The overall conformation of the protein after fitting the core and gate domains separately is more similar to the inward-occluded AtBOR1 (Fig. 7C), rather than the outward-facing hNBCe1 or hAE1 (Fig. 7D,E), indicating that OsBOR3 is in the inward facing conformation. Indeed it is possible that the OsBOR3 is in a slightly more inward open conformation than AtBOR1 although at this low resolution it is difficult to be confident.

## Discussion

The BOR proteins, and their BOT1 homologue from barley^17^, are crucial for efficient uptake and distribution of boron, as well as regulation of intracellular boron concentrations in plants. Here we used a combined approach incorporating mutagenesis in both yeast and plants, together with structural analysis in order to explore in more detail the mechanism of action of these important proteins. A series of AtBOR1 variants were generated based on (i) a known loss of function mutant identified in the ScBOR1p (D311A) and (ii) disease causing mutations in the related SLC4 protein, hAE1 (S466R and A500R). We also included a mutation, P362G, which had no significant effect on the expression, trafficking or function of the transporter.

The mutagenesis studies in yeast and plants were carried out using the AtBOR1 protein while the structural analysis was carried out on the OsBOR3 protein. Use of *Arabidopsis thaliana* for mutagenesis allowed us to generate plant variants on a reasonable timescale, something that would not have been possible using the OsBOR3 protein and rice plants. However, it is clearly important that we study the rice variants of these proteins given the importance of rice as a staple food for large swathes of the world’s population. In our hands, we found the C-terminally truncated OsBOR3_∆1–642_ protein to be an extremely stable BOR variant^38^.

The AtBOR1 D311A is equivalent to the ScBOR1p D347A mutant. In the paper describing the AtBOR1 structure, the authors used the yeast ScBOR1p as a substitute for exploring the potential role of specific residues in substrate binding and transport in a functional complementation assay using a yeast ∆*bor1* strain^25^. They reported that transformation with AtBOR1 failed to functionally complement the ScBOR1p borate efflux activity, as had an earlier study^16^. However in our hands we consistently saw that WT AtBOR1 was able to effectively complement ScBOR1p activity, in agreement with a recent study^39^, thus allowing us to directly assess the effects of mutations in the plant transporter. In agreement with the earlier study on the ScBOR1p, here we report that mutation of AtBOR1 Asp311 to Ala results in a loss of borate efflux activity. Importantly however we also show for the first time what effect this mutant has on plant growth under boron restricted conditions. It is clear that the D311A plant variant exhibits very poor growth in soil, as poor as the *bor1-3* KO plants. Importantly, this variant expresses to a similar level to WT, traffics to the membrane and can be solubilised effectively into detergent based solution indicating that this loss of function is not due to reduced expression or the expression of incorrectly folded protein. These data confirm that this Asp residue which is conserved among BOR proteins is crucial for function. D311A is located in the TM8 which forms part of the core domain. Comparison with the structures of other SLC4 proteins, hAE1 and hNBCe1, reveals that this residue lies in close proximity to the likely substrate binding site between the two half helices, TM3 and TM10. Thus, this residue may have a role in direct substrate binding or may be the proton binding residue critical for energising the transport process. It is also possible that this residue has key roles in both substrate and proton binding.

hAE1 is by far the best characterised of the SLC4 transporters. This protein is expressed in large quantities naturally in erythrocytes and functions as a bicarbonate/Cl^−^ exchanger increasing the CO_2_ carrying capacity of the blood^40^. Mutations in this protein are associated with a wide range of blood diseases^41^. These include the S762R (S466R in AtBOR1) and G796R (A500R in AtBOR1) individual mutants characterised by a loss of anion exchange activity and leakage of monovalent cations through hAE1, responsible for hereditary forms of stomatocytosis^34,35^. Mapping of these mutations onto the structure of hAE1 indicated that the S762R mutation, located at the N-terminal end of TM11, reduces the interactions between TM10 and TM11, TM11 and TM1, and TM11 with a loop connecting to TM3^22^. The G796R mutation located in the middle of TM12 is suggested to alter helix packing. Thus, both mutations are likely to affect the overall structural stability of the protein, although this has not been definitely demonstrated. We assessed what effects the equivalent mutations have on the expression, localisation and activity of AtBOR1. The S466R and A500R AtBOR1 variants express in yeast although both exhibit markedly lower expression than the WT transporter. Importantly, both variants do localise at the plasma membrane, suggesting that at least some of the protein is correctly folded and trafficked. FSEC and hFSEC analysis were not possible for the A500R mutant. This is most likely due to the low expression level of this protein, however we cannot rule out that low stability of this protein means that it doesn’t got into detergent based solution but rather aggregates upon extraction from the membrane^42^. In contrast, the S466R mutant was extracted into DDM-based solution effectively, although from the hFSEC analysis is less stable than the WT protein. Borate efflux analysis revealed that both mutants exhibit a loss of transporter activity similar to the D311A mutant and this is further clearly illustrated by the poor growth of the S466R and A500R plant mutants grown in both soil and on media under boron restricted conditions. It is possible that some of the loss of function for these mutants, and particularly the A500R, is due to low expression of these proteins. In the case of the hAE1 mutants it is clear that fundamental changes within the protein abolish bicarbonate/Cl-exchange and induce cation leak^34,35^. Given the structural similarity of the BOR proteins and hAE1 it is likely that the mutations have similar effects at the molecular level.

Our structure confirms findings from earlier studies on other homologous proteins that OsBOR3 is dimeric with each protomer organised into core and gate domains. In our case the core domain was slightly less well resolved compared to the gate domain, most likely as a result of the elevator motion carried out by this domain as part of the transport cycle. We did not lock our protein through the introduction of mutations or the use of a conformation specific antibody, but either of these approaches might facilitate obtaining a higher resolution structure.

In summary we have demonstrated that AtBOR1 mutations, equivalent to disease causing mutations in hAE1, result in markedly reduced plant growth phenotypes.

## Materials and methods

### Expression, purification and cryo-EM grid preparation

The proteins were expressed and purified as previously described^33,38^. In brief, for cryo-EM analysis a protein lacking amino acid residues 643–672 and incorporating a Q228T mutation was introduced to remove a naturally occurring TEV cleavage site (using oligomers: O_SB3_Q-T_F 5′-tctgagccaccccgtaccatacacccatgaccttgccc-3′ and O_SB3_Q-T_R 5′-gggcaaggtcatgggtgtatggtacggggtggctcaga-3′) was used. The protein, referred to as OsBOR3_∆1–642_^38^, was expressed as a fusion protein with C-terminal yeast enhanced GFP and 8 His tags and incorporating a TEV cleavage site^43^. The protein was expressed in FGY217 *Saccharomyces cerevisiae* cells^44^ in a typical culture volume of 12 L -URA medium supplemented with 0.1% (w/v) glucose incubated at 30 °C. The -URA media was prepared with 6.7 g/L Yeast Nitrogen Base without amino acids (Sigma-Aldrich, Y0626-1 kg) and 2 g/L of amino acid mix [prepared in house by mixing: 18 mg/L Adenine Hemisulfate (A9126), 76 mg/L Alanine (A7627), 76 mg/L Arginine hydrochloride (A5131), 76 mg/L Asparagine monohydrate (A8381), 76 mg/L Aspartic acid (A8949), 76 mg/L Cysteine hydrochloride monohydrate (C7880), 76 mg/L Glutamic acid monosodium salt (G1626), 76 mg/L Glutamine (G3126), 76 mg/L Glycine (G7126), 76 mg/L Histidine (H8000), 76 mg/L myo-Inositol (I5125), 76 mg/L Isoleucine (I2752), 380 mg/L Leucine (L8000), 76 mg/L Lysine monohydrochloride (L5626), 76 mg/L Methionine (M9625), 8 mg/L p-Aminobenzoic acid potassium salt (A0254), 76 mg/L Phenylalanine (P2126), 76 mg/L Proline (P0380), 76 mg/L Serine (S4500), 76 mg/L Threonine (T8625), 76 mg/L Tryptophan (T0254), 76 mg/L Tyrosine disodium salt (T2269), 76 mg/L Valine (V0500). All the products were purchased by Sigma-Aldrich. Addition of galactose, to a final concentration of 2% (w/v), was used to induce protein expression once the culture reached an OD_600_ = 0.6. Protein expression was induced for 22 h and the cells harvested by centrifugation (5000×*g* for 10 min at 4 °C) followed by storage at − 80 °C until further use. A 2 day protocol was used for preparation of cryo-EM grids including membrane preparation, protein isolation and grid preparation and freezing. The cells (from 4 L of culture) containing OsBOR3_∆1–642_ were thawed and then resuspended on ice in 50 mM Tris (pH 7.5), 1 mM EDTA, 0.6 M sorbitol) supplemented with cOmplete™ EDTA free protease inhibitor tablets (Roche). The cells were lysed using a Constant Systems Cell Disrupter and membranes harvested by differential centrifugation (10,000×*g* for 10 min followed by 100,000×*g* for 1 h at 4 °C). The membranes were immediately resuspended into solubilisation buffer (1 × PBS (pH 7.5), 100 mM NaCl, 10% glycerol (v/v), 1% LMNG (w/v)) supplemented with protease inhibitor tablets (Roche), followed by incubation at 4 °C for 1 h. Insoluble material was removed by ultracentrifugation (100,000×*g* for 1 h at 4 °C) and the OsBOR3_∆1–642_ in the soluble extract was purified using a three step process using LMNG at a concentration of 1× CMC; His-trap at a flow rate of 1 mL/min, TEV cleavage followed by reverse His-trap and size exclusion chromatography at a flow rate of 0.5 mL/min. The protein was eluted from the SEC column in 20 mM Tris (pH 7.5), 150 mM NaCl, 0.75× CMC LMNG. The single SEC fraction containing the highest concentration was used to prepare the grids directly with no further buffer exchanges or concentration steps. The protein was also analysed by Coomassie stain SDS-PAGE.

### Negative stain TEM

Protein preparations were screened by negative-stain EM to assess for monodispersity and overall quality of the protein preparation prior to cryo-EM analyses. For negative strain EM analysis, 0.01 mg/mL OsBOR3_Δ1–642_ in a total volume of 3 μL was applied onto freshly glow-discharged carbon coated 400 mesh grids (Agar scientific), excess solution removed by blotting from the side and stained with 2% (w/v) uranyl acetate. Negative stain EM micrographs were recorded on a Tecnai T12 microscope at 120 kV using an FEI 2 K eagle camera.

### CryoEM grid preparation and data collection

300 mesh Cu Quantifoil R2/2 grids (Agar Scientific) were glow discharged for 90 s prior sample deposition. 3 µL OsBOR3 (1 mg/mL) was applied to the grid, blotted and then plunged into liquid ethane using a Vitribot mark IV (Thermo Fisher Scientific) at 4 °C, 100% humidity, 3 s blot time and -2 blot force. Plunge frozen grids were stored under liquid nitrogen until use.

EPU automated imaging software (Thermo Fisher Scientific) was used to collect electron micrograph movies on a 300 keV Titan Krios (Thermo Fisher Scientific) fitted with a K3 direct detection camera (Gatan) in super resolution mode. All collection parameters are shown in Supplementary Table S3.

### CryoEM data processing and density reconstruction

The collected micrograph movie frames were aligned using MotionCor2^45^ and subsequently the CTF parameters were estimated with CTFFind4^46^. All the micrographs were then assessed by eye for quality using the FFT spectra to discard micrographs with excessive image drift or crystalline ice. Unless otherwise stated all the following data processing was carried out in RELION^47^. Particles were picked manually and with the RELION auto-picking function and then sorted by their similarity to the reference 2D classes (Z-score). Several rounds of 2D classification were used to select a range of orientations before generating an initial model low pass filtered to 15 Å. A three class 3D classification was performed with the initial model to select a homogenous particle set for refinement. The class with the highest particle occupancy and resolution was taken forward for refinement. 3D refinement with C2 symmetry imposed, followed by post-processing yielded a reconstructed density of between 5 and 6 Å estimated global resolution with a B-factor of − 220 Å^2^. Details of the mask used are available from the Electron Microscopy DataBank (EMDB) under the accession code EMDB-11996. All maps were visualised in 3D using UCSF Chimera^48^.

### Mutagenesis

AtBOR1 point mutants were created with the QuikChange Lightning Site-DirectedMutagenesis Kit (Agilent Technologies), using as template DNA, wild-type or AtBOR1 in the pDDGFP2 vector. The oligonucleotides used are shown in Supplementary Table S1.

### Functional complementation analysis

pDDGFP2 vectors containing the genes encoding WT AtBOR1, WT OsBOR3 and OsBOR3_∆1–642_ were transformed into a *S. cerevisiae* ∆*bor*1p strain for functional complementation analysis as previously described^33^. As a negative control empty pDDGFP2 vector was also transformed into the *S. cerevisiae* ∆*bor*1p strain. Transformants were incubated in 10 mL -URA with 2% (w/v) glucose at 30 °C and 300 rpm shaking overnight. A fivefold serial dilution was carried out with cells starting at OD_600_ 0.5 in -URA media. 10 μL of each dilution was spotted on -URA plates with 2% (w/v) galactose and supplemented with the following concentrations of boric acid: 0, 5, 7.5 mM. Plates were incubated for 30 °C for 5 or 7 days and imaged using a ChemiDoc MP Imaging System (Bio-Rad).

### FSEC and hFSEC

AtBOR1 proteins were expressed as described above and membranes containing the different AtBOR1 proteins were diluted in solubilisation buffer (PBS (pH 7.4), 1% (w/v) DDM, supplemented with 1 tablet of protease inhibitor) to give a final total protein concentration of 50 μg/mL and incubated with gentle rocking at 4 °C for 1 h. Insoluble material was removed by centrifugation at 14,000×*g* for 1 h at 4 °C. The solubilisation efficiency of each protein was calculated as the proportion of the initial GFP fluorescence that remained after solubilisation. 500 μL supernatant was injected onto a Superose 6 10/300 column equilibrated with 20 mM Tris (pH 7.5), 150 mM NaCl and 0.03% (w/v) DDM. The elution was collected, from 6.4 mL elution volume (void), in 200 μL fractions in a clear bottomed 96-well plate. The GFP fluorescence of each fraction was measured using a SpectraMax M2e with an excitation wavelength of 488 nm and an emission wavelength of 512 nm. For heated FSEC experiments, the solubilised protein was incubated at 46 °C for 10 min and any insoluble aggregates removed by a 10 min centrifugation (14,000×*g* at 4 °C) prior to loading onto a Superose 6 10/300 column. The samples were collected and analysed as described above.

### Plant genotypes

Using *Agrobacterium tumefaciens*-mediated transformation, the pBOR1::BOR1-GFP proteins containing the respective point mutations were introduced into the *bor1-3 Arabidopsis thaliana* background, which carries a T-DNA insertion in the AtBOR1 gene (SALK_037312^49^). To select for plants carrying the desired transgene, T1 seeds were grown on half strength Murashige and Skoog (MS) medium containing 50 mg/L kanamycin. The presence of the respective BOR1 mutation in each individual line was verified by PCR on genomic DNA (primers 5′-ATGCTTGATGTTCCAATCGTC-3′ and 5′ AGCTCCTCGCCCTTGCTCAC-3′) followed by sequencing. The resulting T2 seeds were analysed for their segregation on kanamycin to avoid lines containing multiple insertions of the construct. At least two independent insertion lines were isolated per construct to minimize the risk of positional effects. The T2 seeds were used for phenotyping experiments.

### Plant growth conditions

For analysis of shoot fresh weight of seedlings grown in axenic culture, plants were grown in square petri dishes (12 × 12 cm) for 3 weeks. Seeds were surface-sterilized and stratified for 2–3 days at 4 °C. Half strength MS basal medium (corresponding to Sigma M5524) was prepared by adding all ingredients except boric acid. The MS medium contained 5 g/L sucrose and 0.5 g/L MES (2-(N-morpholino)ethanesulfonic acid) and was adjusted to pH 5.7 with KOH. To remove residual boric acid the medium was treated overnight with B chelator Amberlite IRA-743 (3 g/L, Sigma). Before autoclaving, boric acid (H_3_BO_3_, Sigma-Aldrich, Catalog number 15663) was added to the medium to a concentration of 30 µM (sufficient B) or 300 nM (low B). The medium was solidified using 4.5 g/L Gelrite (Duchefa). The seeds were germinated vertically on medium containing 50 mg/L kanamycin to select T2 seedlings that carry the desired transgene. When 5 days old, resistant seedlings were transferred to fresh medium with either low or sufficient boron but without kanamycin and grown horizontally thereafter. When the plants were 3 weeks old the shoots were separated from the root system and weighed. Plates were kept in a growth chamber set to 22 °C day/20 °C night temperature under 12 h of light.

To determine plant phenotypes in soil culture, seedlings were initially grown vertically on half strength MS medium containing 30 µM boron and 50 mg/L kanamycin as described above. After 1 week, 10–12 resistant seedlings per genotype were transferred to soil into individual pots (5 cm). After transfer to soil, the plants were moved to an Aralab growth room set to 22 °C day/20 °C night temperature under 12 h of light at 60% relative humidity. The plants were watered moderately and the rosette fresh weight was analysed at 5 weeks of age.

### Boron transport assay

Synthetic genes of AtBOR1 mutants in pDONR221 were synthesized by Genescript (https://www.genscript.com/). The synthetic genes were subcloned into yeast expression vector pDDGFP2 and transformed in to FGY217 (Δbor1) yeast strain as described by^33^. Borate efflux assay in yeast cells was performed as described in Takano et al.^14^ with some modifications. Briefly, yeast cells harvested at mid-exponential phase (OD_600_ ~ 0.6). were transferred to the synthetic medium (pH 5.5) containing 1 mM of boric acid and incubated for 1 h. After 1 h cells were washed with ice cold distilled water and harvested by centrifugation at 3000×*g* for 2 min at 4 °C. To release the intracellular boron the harvested cells were boiled at 90 °C for 30 min and the supernatant was used to measure the boron content by using a curcumin based colorimetric protocol based on absorbance values at 550 nm^50^. The coumarin efflux assay results were normalised using GFP flourcsence; 100 µL of each batch of cells were aliquoted into the microplate and absolute fluorescence was quantified using a Fluorescence Microplate reader with excitation max 488 nm and emission max 509 nm. The absolute fluorescence was normalized to WT to obtain the fold GFP expression.

### Confocal microscopy

Yeast cells expressing AtBOR1-GFP were harvested at mid-log phase (OD600 ~ 0.6). 100 µL of these cells were aliquoted in microplate and absolute fluorescence was quantified using Fluorescence Microplate reader with excitation max 488 nm and emission max 509 nm. To assess the localization of AtBOR1-GFP, yeast cells expressing the AtBOR1 proteins were harvested at mid-log phase and analysed using Leica SP5 confocal fluorescence microscope with the excitation and detection wavelengths of 488 nm and 500–540 nm, respectively.

### Plant research compliance statement

We obtained our seeds, with permission, from colleagues Japan in compliance with the Nayoga protocols. No seeds were collected from the wild and no licences were required.

## Supplementary Information

Supplementary Information.

## Data Availability

The final cryoEM density map of OsBOR3 has been deposited to the Electron Microscopy DataBank (EMDB) under the accession code EMD-11996.
