# Supplementary material for: Structural and functional insights into the mechanism of action of plant borate transporters
Source: Sci Rep. 2021 Jun 10;11:12328. doi: 10.1038/s41598-021-91763-6 (PMC8192573; doi:10.1038/s41598-021-91763-6)
Supplement: Supplementary file 1 — Supplementary Information. [file 41598_2021_91763_MOESM1_ESM.docx]

**Supplementary Information,**

**Saouros et al. Structural and functional insights into the mechanism of action of plant borate transporters**

**Supplementary Table 1**

**Oligonucleotide primer sequences used to generate the AtBOR1 mutants.**

| **Construct** | **Oligonucleotide (5’ - 3’)** |
| --- | --- |
| AtBOR1 D311A | F: ctgtgctttactacttcGCCcatagtgtagcttcaca  R: tgtgaagctacactatgGGCgaagtagtaaagcacag |
| AtBOR1 P362G | F: cggtgtcattcctcaatctGGAatgcataccaagagcttagc  R: gctaagctcttggtatgcatTCCagattgaggaatgacaccg |
| AtBOR1 S466R | F: agtcaaagaacaacgggtaCGCaacttgcttcagtc  R: gactgaagcaagttGCGtacccgttgttctttgact |
| AtBOR1 A500R | F: ggctattttgccttcatgCGCatcgaaagcttacccgg  R: ccgggtaagctttcgatGCGcatgaaggcaaaatagcc |

**Supplementary Table 2**

**Expression levels and solubilisation efficiencies of the AtBOR1 mutants**

| **Construct** | **Expression level (mg / L)** | **Solubilisation efficiency* (%)** |
| --- | --- | --- |
| WT | 2.7 | 79 |
| D311A | 2.3 | 74 |
| P362G | 2.5 | 66 |
| S466R | 1.7 | 53 |
| A500R | 0.7 | 29 |

*Efficiency of solubilisation into 1% DDM, expressed as a % of the total fluorescence of membranes expressing the individual constructs prior to solubilisation.

**Supplementary Table 3**

**CryoEM data collection parameters**

| **Data collection parameters** |  |
| --- | --- |
| Microscope | Titan Krios |
| KeV | 300 |
| Camera | K3 direct detection |
| Collection mode | Super-resolution |
| C2 aperture (μm) | 50 |
| Objective aperture (μm) | 70 |
| Nominal magnification | 81 000 |
| Pixel size (Å) | 1.1 |
| Number of frames | 38 |
| Dose rate (e^-^/Å^2^/s) | 12.40 |
| Total dose (e^-^/Å^2^) | 50 |
| Defocus range (μm) | -1.2 to -4.0 |
| Number of micrographs | 6285 |

| **Data processing statistics** |  |
| --- | --- |
| Initial particle number | 2 548 682 |
| Particle number after sorting | 629 716 |
| Final particle number | 116 140 |
| Symmetry imposed | C2 |
| Global map resolution (Å) | 5.1 at FSC = 0.143 |
| Local resolution range (Å) | 5.0 - 6.0 |

**Supplementary Figure 1**

**
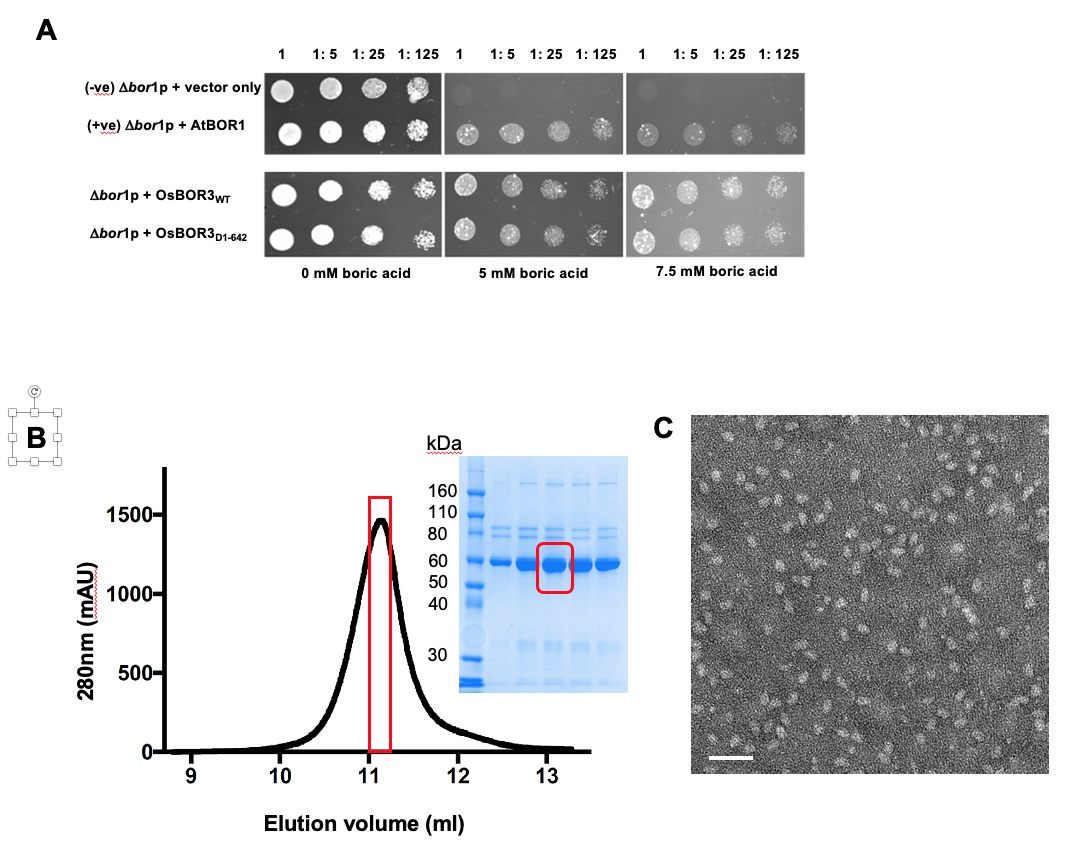
**

**Supplementary Figure 1: Sample characterization and cryoEM images of OsBOR3_Δ1-642_. A)** Functional complementation analysis of OsBOR3_WT_ and OsBOR3_Δ1-642._ Δ*bor1p* *S. cerevisiae* cells were spotted in a 4× series of dilution from left to right on media containing 0, 5, or 7.5 mM boric acid. Plates were incubated at 30 °C for 5 days before being imaged. FGY217 Δ*bor1p* cells were transformed with an empty vector as a negative control (Δ*bor1p* + pDDGFP). Δ*bor1p* cells overexpressing AtBOR1 were also analyzed as a positive control. **b)** SEC profile of the purified OsBOR3_Δ1-642_. The single symmetrical peak corresponds to the OsBor3_Δ1-642_ dimer. The fraction with the highest mAU value and therefore the highest concentration of protein was used for EM). The inset shows the SDS-PAGE analysis of the key fractions of the protein sample (kDa indicates the molecular weight markers). The fraction used for EM analysis is indicated in the red boxes on both the SEC profile and the gel. **c)** A representative negative stained micrograph of OsBOR3_Δ1-642_ (scale bar = 40 nm) collected on a Tecnai T12 microscope at 120 kV using an FEI 2K eagle camera. *Please see Supplementary Figure 4 for the raw gel image and the unlabeled photographs for the Functional Complementation analysis.*

**Supplementary Figure 2**


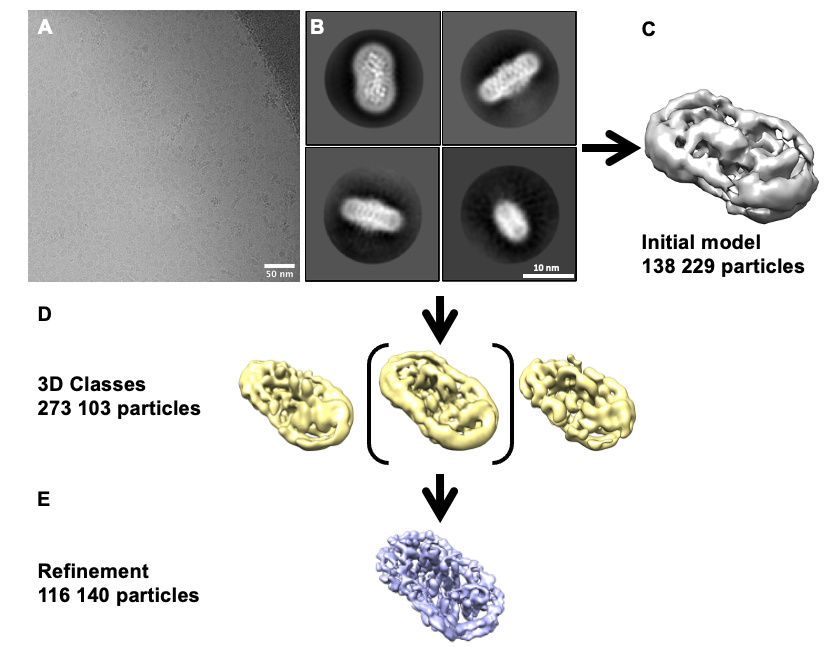


**Supplementary Figure 2 CryoEM processing workflow for OsBOR3_Δ1-642_ density reconstruction. A**) A representative cryoEM micrograph of OsBOR3_Δ1-642_ (scale bar = 50 nm) collected on a Titan Krios microscope operated at 300 kV on a Falcon III direct electron detector. **B**) Four 2D classes generated after several rounds of particle classification (scale bar = 10 nm). **C**) An initial model was reconstructed from a subset of particles extracted from 2D classifications. **D**) These 3D classes (khaki) enabled us to select the best quality particles from the highest resolution class which clearly shows separate helices in the core of the particle. **E**) The refined and post-process filtered density reconstruction (light purple) was made from 116 140 particles.

**Supplementary Figure 3**


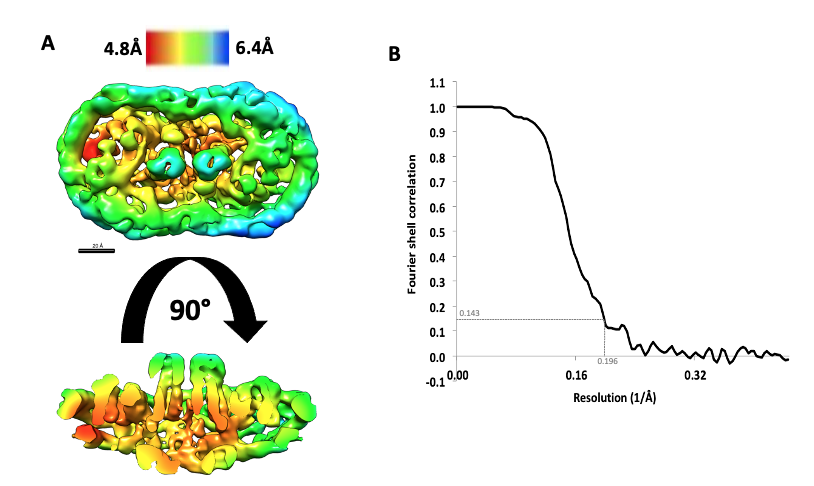


**Supplementary Figure 3: OsBOR3_Δ1-642_ map resolution A**) A local resolution filtered map color coded according to resolution which ranges from 4-6 Å (scale bar of 20 Å). The top image is viewed from the extracellular side of the membrane whilst the bottom image shows a 90° rotation about the x axis with a cut away to show the center of the dimer. **B**) Mask-corrected Fourier shell correlation (FSC) curve produced from unfiltered half-maps using RELION. The data in this figure result directly from the final filtered map shown in Supplementary Figure 2E.

**Supplementary Figure 4**

**
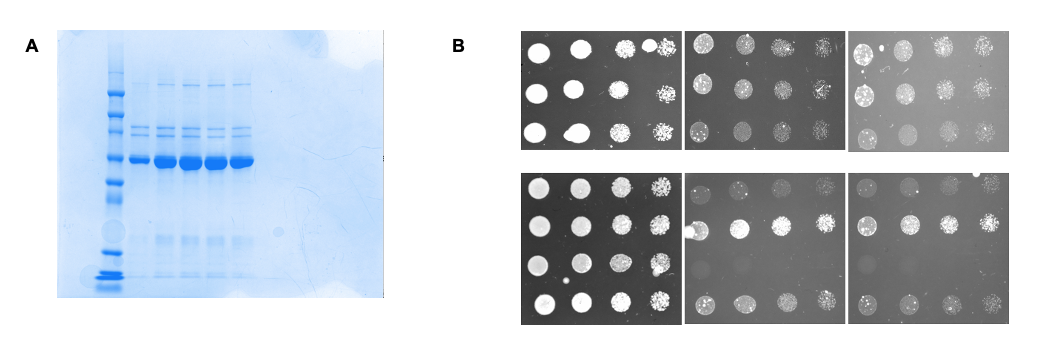
**

**Supplementary Figure 4** **A)** The raw gel image and **B)** the unlabeled photographs for the Functional Complementation analysis as shown in Supplementary Figure 1
